# Optical Properties of Thomson Scattering Diagnostics Lower Window Glass under Laser Irradiation

**DOI:** 10.3390/ma14112702

**Published:** 2021-05-21

**Authors:** Jian Zhou, Qing Zang, Junyu Zhao, Shumei Xiao, Yong Che, Mengfang Ren

**Affiliations:** 1 School of Materials and Chemical Engineering, Anhui Jianzhu University, Hefei 230601, China; jianzhou@ipp.ac.cn; 2 Institute of Plasma Physics, Chinese Academy of Sciences, Hefei 230031, China; zhaoj@ipp.ac.cn (J.Z.); smxiao@ipp.ac.cn (S.X.); cheyong@ipp.ac.cn (Y.C.); mfren@ipp.ac.cn (M.R.)

**Keywords:** Thomson scattering, laser irradiation, glass, transmittance

## Abstract

Tokamak diagnostic window glass is an indispensable optical medium in fusion research. The transmittance of the device affects the optical performance and accuracy of the diagnostic system. Especially, the window glass serves as the entrance of the light source while performing the sealing function for the active diagnosis method represented by Thomson scattering diagnostics. In this work, we studied the influence of the laser irradiation and tokamak discharge on the EAST (Experimental Advanced Superconducting Tokamak) Thomson scattering diagnostic borosilicate glass window. Using X-ray photoelectron spectroscopy (XPS) and Raman scattering, we found that carbon-based impurities in the device aggravated the film damage due to laser irradiation, reducing the performance of the coating of the glass. Besides, the laser and the various rays of tokamak discharge generated many point defects in the glass, increasing the light absorption of the glass. These two factors caused the glass transmittance to drop significantly (from 99.99% to 77.62%). In addition, the long-term laser irradiation primarily reduced the transmittance, while environmental rays had a minor impact on the same. This work provides valuable insights into the selection and effective use of glass in optics-based diagnostics.

## 1. Introduction

An all-superconducting noncircular tokamak EAST is a ring-shaped magnetic confinement device for studying fusion energy. Through the development of multiple confined plasma operating modes in the device, a solid theoretical foundation is laid for the realization of magnetic confinement fusion in the future. The real-time control and theoretical research of tokamak physical experiments are inseparable from various plasma operation parameters, such as plasma current, ion temperature density, and electron temperature density. As the interactive channel between the diagnostic system and plasma, window glass is used in various diagnostic systems, such as Thomson scattering, polarization interferometer, far-infrared interferometer, charge exchange compound spectroscopy, and curved crystal spectrometer. During the experiment, the optical performance of the glass directly affects the diagnosis accuracy. Studying the physical properties of glass under different experimental conditions and obtaining the law of change of window glass under laser and irradiation conditions are necessary for improving diagnostic accuracy, eliminating potential hazards in fusion research, and ensuring the safe operation of the tokamak device [1,2,3].

In this work, the influence of EAST Thomson scattering diagnostic window glass on the diagnostic performance of the device was studied. Given the actual conditions of the EAST device, the size requirements of the enclosed vacuum, and the requirements to ensure the performance of the diagnosis itself, the glass requires high transmittance, high radiation resistance, and high laser damage threshold. Quartz, borosilicates, and glass-ceramics can meet these requirements, while high-borosilicate glass offers both affordability and practicality [4,5]. High-borosilicate glass has low dispersion and refractive index and good light transmission performance, which can be applied to many optical window applications [6].

During the operation of EAST, the glass receives long-term repeated laser irradiation, such as polarization interferometer or Thomson scattering. Ying Du et al. [7] showed that long-term high-frequency laser irradiation can damage the coating material. After the coating material absorbs the laser energy, delamination takes place, accumulating stress under repeated laser exposures and seriously affecting the performance of the coating [8]. For the glass, repeated pulses can cause internal defects, the color center and broken bonds can continue to increase, and alkali metal cations form defects of various characteristics under the influence of laser and radiation. Boizot found that quartz containing Zr_2_O_3_ Zr^3+^ defects was formed by irradiation [9]. MoO_2_ in glass-ceramics formed Mo^5+^ defects [10]. Ollier et al. [11] studied TiO_2_-containing borate glass exposed to different layers. Ti^4+^ or Ti^3+^ reduction process under high radiation conditions destroyed the internal structure of the glass, resulting in an irreversible drop in transmittance [12]. The glass used in this work was a window glass experimented in the winter of 2019. The transmittance curve of the glass at different positions after the experiment was measured by a spectrophotometer, and the glass was compared with the well-coated glass. Using a high-magnification microscope, X-ray photoelectron spectroscopy (XPS) was used to analyze the film surface and Raman scattering and electron paramagnetic resonance (EPR) to analyze the glass structure and study the laser irradiation in single-round experiments. The difference between the research content of this article and other radiation on the optical properties of glass is that the impurities and radiation generated during the operation of the Tokamak have an adverse effect on the coated glass, which greatly reduces the service life of the window glass.

## 2. Materials and Methods

**Figure 1:**
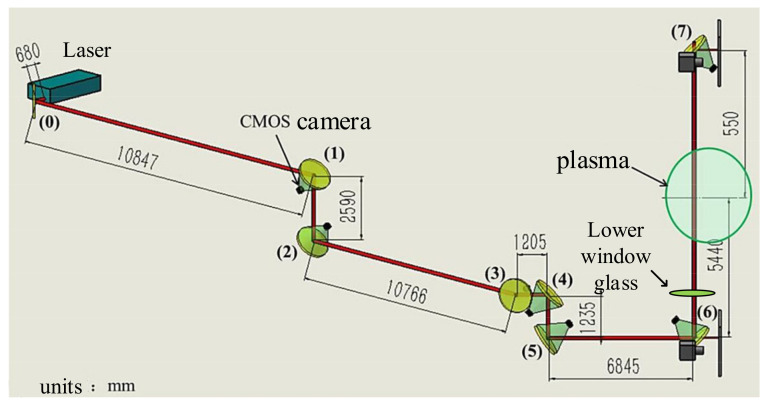
EAST Thomson scattering diagnostic optical system of Laser transmission light path.

**Figure 2:**
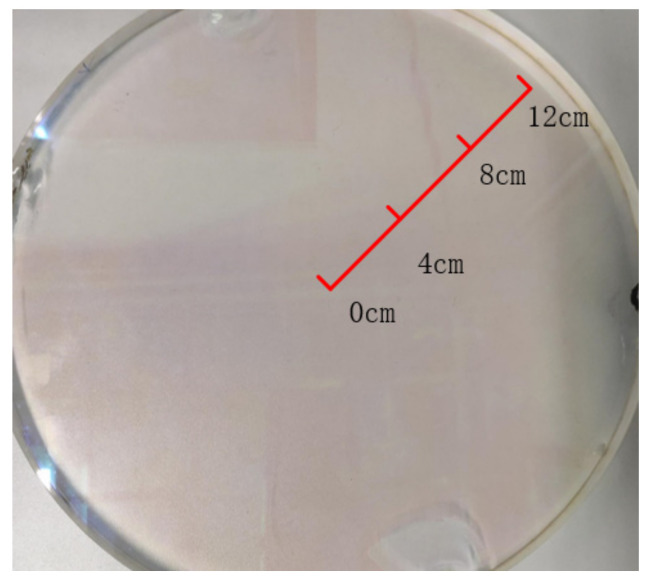
Window glass under Thomson scattering diagnosis, 28 cm in diameter and 2 cm in thickness.

The laser used for Thomson scattering diagnostics was combined with mirrors to transfer the laser light from the laboratory to the window of the EAST device. The laser used in the laboratory is ND: YAG laser, and its specific parameters are shown in Table 1. The EAST Thomson scattering optical system is shown in Figure 1. The glass size of the Thomson scattering window was 280 mm in diameter and 20 mm in thickness. The surface was coated with an antireflection coating to ensure that it had a transmittance of 99.999% at 1064 nm. The main component of the film was H_f_O_2_. The glass composition is shown in Table 2. Figure 2 shows the window glass. The glass surface was cleaned before the experiment and the glass on the EAST window was fixed with a vacuum flange. It was exposed to an electrical discharge, during which some areas were irradiated by lasers. When the tokamak is discharging, it will produce neutrons, gamma rays, X-rays, etc. These high-energy rays and radiation will also affect the glass material to a certain extent. The radia-tion power density of the plasma (infrared-soft X-ray band) is about 10–100 kw/m^3^. The density of the boundary plasma is about 1–2 × 10^−19^ m^3^. However, the length of the lower neck tube is about 2.7 m, and the diameter of the hole connecting with the vacuum chamber is about 15 cm. When the plasma ruptures, a large number of escaped electrons will hit the vacuum wall, and only a very small amount of particles will pass through the holes of the divertor and reach the lower neck tube through the lower window. Neutrons and gamma rays, which have a serious impact on glass materials, during radio frequency wave or ohmic discharge, the neutron flux is 5.0 × 10^3^ cm^−2^s^−1^; 4.0 × 10^5^ cm^−2^s^−1^ during lower power NBI discharge; 3.5 × 10^6^ cm^−2^s^−1^ at higher power NBI discharge. The highest measured value of gamma rays around the device environment during NBI discharge is 2080 µSv/h.The window glass studied in this work started from the vacuum sealing in September 2019, and the experiment ended in January 2020. The number of experimental discharges was about 7000 shots. As shown in Figure 1, After seven mirror reflections, the laser consumes a part of the energy during the transmission process. The average energy of the laser measured in front of the lower window glass is 2.4 J. The amount of radiation accumulated in a discharge time of about 150 h is relatively small. It can be considered that the radiation accumulated in a round of experiments has a smaller impact on the window glass than the hundreds of thousands of laser irradiations with an average energy of 2.4 J.

After the experiment, the surface coating was observed and analyzed by a high-power microscope and XPS. We re-polished the glass with the same parameters as before the glass coating. The transmittance was measured with a spectrophotometer at different positions before and after the coating was polished, and the polished glass was analyzed by Raman scattering and EPR. Its B class of Stripe degrees, Level 2 of optical uniformity, Level 3–4 of surface roughness and plane consistency of PV value.

## 3. Results

### 3.1. Glass Transmittance

**Figure 3:**
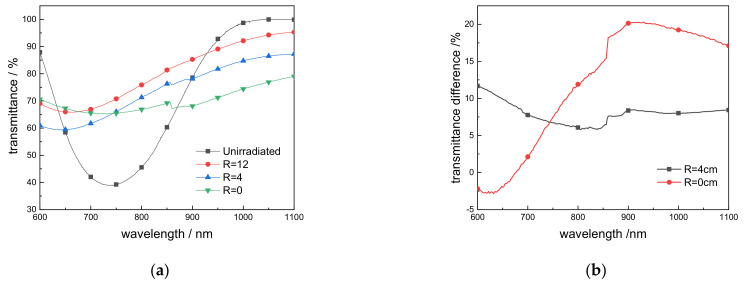
(**a**) Transmittance at glass R = 0 cm, R = 4 cm, R = 12 cm and unirradiated coated glass; (**b**) Transmissivity difference ΔT = (T_R = 12_ − Ti)/T_R = 12_. i = 0 cm, 4 cm.

After a round of experiments, three positions were randomly selected: the center position (R = 0 cm) and two other positions (R = 4 cm and R = 12 cm), as shown in Figure 2. The transmittance was measured and compared based on the sample position on the glass, as shown in Figure 3a,b.

Compared with the 99.99% transmittance of the intact coated glass at 1064 nm, after long-term experiments, the edge of the window glass (R = 12 cm) still had 94.62% transmittance at 1064 nm. At the middle position (R = 4 cm), the transmittance dropped to 86.79%, while it was only 77.61% at R = 0 cm. Compared with the intact coated glass, the transmittance difference at the edge position (R = 12 cm) was 5.44%, while it was 13.26% at the middle position (R = 4 cm) and 22.43% at the center position (R = 0 cm), showing that the transmittance has decreased to varying degrees. At the same time, the differences in transmittance between the center position R = 4 cm and the center position R = 0 cm compared to the edge position were 8.26% and 17.97%, respectively.

**Figure 4:**
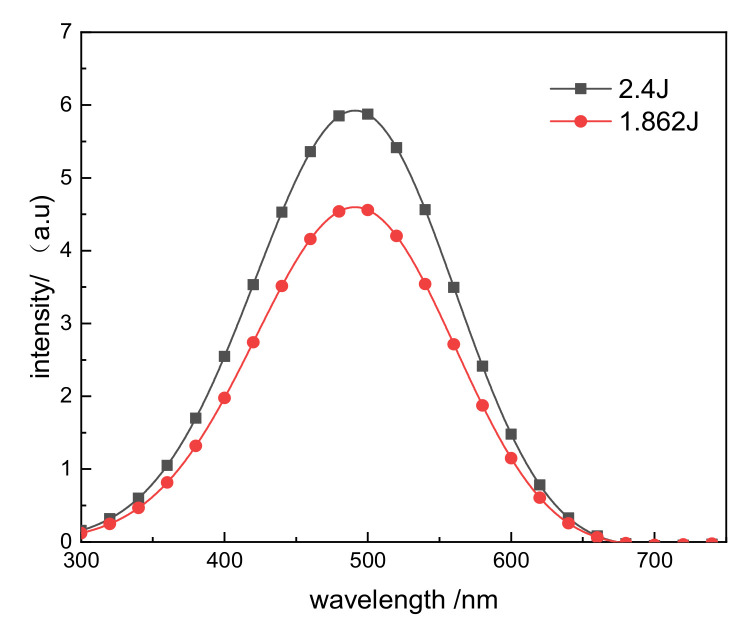
Thomson scattering spectrum generated by the laser when the coating was intact versus damaged.

As shown in Figure 1, when the laser is transmitted to the front of the lower window glass, its average energy is 2.4 J. When the glass film layer maintains a transmittance of 99.999%, 2.4 J energy can completely enter the vacuum chamber, but after the film layer is damaged, when the transmittance drops to 77.61%, the laser energy is only 1.862 J. When the electron temperature inside the device is 5 keV, the Thomson scattering spectrum formed after the laser enters the vacuum chamber after the film is intact and damaged is shown in Figure 4. It means that the resulting density measurement error exceeded 20%, which substantially impacted the diagnostic accuracy.

**Figure 5:**
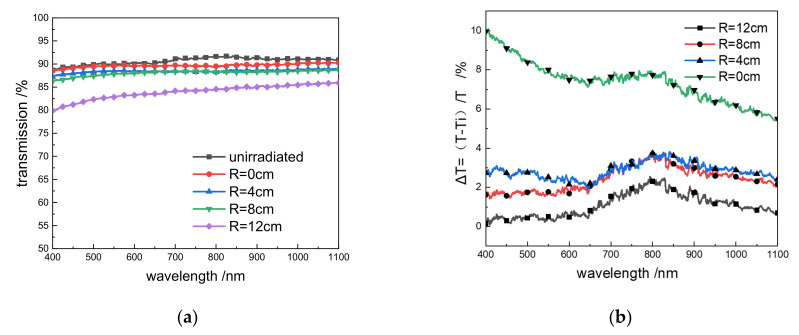
(**a**) After polishing, R = 0 cm, R = 4 cm, R = 8 cm, R = 12 cm and the transmittance of unirradiated glass after polishing; (**b**) The ratio of the transmittance between different positions of the glass and the unirradiated glass ΔT = (T − Ti)/T, T is the transmittance of unirradiated glass.

We selected a new window glass with no coating on one side and tested the transmittance of the re-polished and polished window glass. After polishing, we selected four positions (R = 0 cm, R = 4 cm, R = 8 cm, and R = 12 cm), and the center of the new window glass. Figure 5a shows the transmittance at different positions of the glass and the nonirradiated glass after polishing, and Figure 5b shows the ratio of the difference in transmittance between various positions.

It can be seen from Figure 5 that, after polishing, the 600–1100 nm transmittance curve became flat, and the overall glass transmittance decreased, and the visible light band dropped significantly, indicating that the glass was exposed to radiation and laser irradiation. The absorption in the visible light band increased. At a laser wavelength of 1064 nm, compared with the 91.06% transmittance of the uncoated new window glass, the transmittances at R = 0 cm, R = 4 cm, R = 8 cm, and R = 12 cm were 85.86%, 87.24%, 88.66%, and 90.23%, respectively, decreasing by 0.9%, 2.25%, 2.63%, and 5.71%. Thus, the drop in the glass transmittance increased as the sample position was closer to the center.

### 3.2. Surface Microscopic Observation

**Figure 6:**
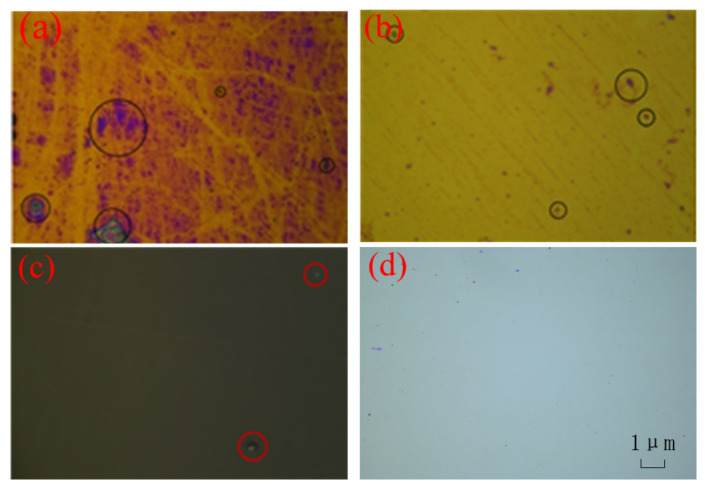
Glass surface at different positions under the microscope: (**a**) R = 0; (**b**) R = 4; (**c**) R = 8; (**d**) R = 12.

Figure 6 shows the glass surface under a high-magnification microscope. At the center, large ablation marks and surface contamination were observed, while relatively small contamination was found at R = 4 cm. At R = 8 cm and R = 12, almost no scars or contamination was obtained. It shows that the dense laser beam caused serious ablation damage on the surface of the H_f_O_2_ film, and a certain degree of contamination appeared on the surface of the film. However, no obvious scars or contamination were found in the locations that received less laser radiation.

### 3.3. XPS

**Figure 7:**
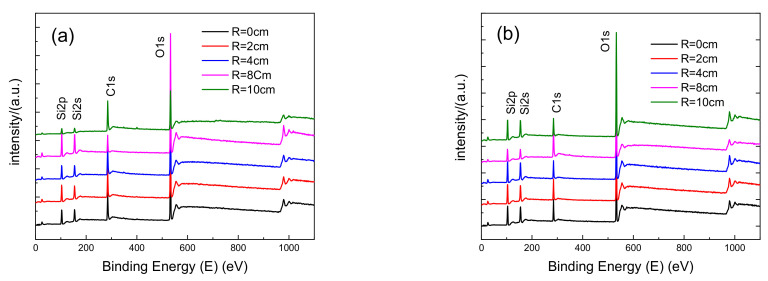
XPS analysis (**a**) of the upper surface, (**b**) underneath the surface.

To further analyze the composition of the contaminants on the film surface, five locations with an interval of 2 cm from the center to the edge were equidistantly selected for XPS analysis. Figure 7 shows the XPS energy spectra of the (a) upper and (b) lower glass surfaces. Most contaminants on the glass surface were C-based. The source of the impurity was the lower divertor in the EAST device. Due to the plasma discharge, the plasma hit the lower divertor, causing it to produce C impurity. The impurity fell on the glass surface through the lower neck tube and then passed for a long time. The laser was irradiated to deposit the C element on the glass surface.

The XPS surface analysis and the surface content ratio showed that at the same location, the upper surface C element content was higher than that underneath the surface (Table 3). As the glass sample position got closer to the center, the surface C ratio and content increased. The closer the upper surface was to the center, more C elements were deposited on the surface of the film. At the same time, the content of C elements in different positions showed randomness. As the main environmental pollution factor, C impurity is deposited on the surface of the film under the action of the laser, which increases the absorption capacity of the film surface to the laser, deepens the accumulated damage to the film, and causes the film performance to decline rapidly.

### 3.4. Raman Spectroscopy

**Figure 8:**
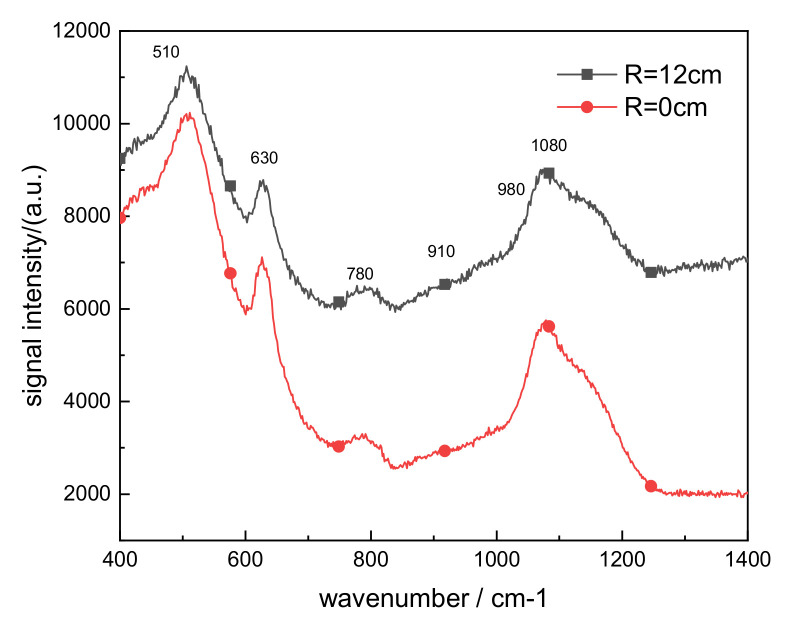
Raman scattering spectra at R = 0 cm and R = 12 cm.

Figure 8 shows the Raman spectra at the center of the glass at R = 0 cm and the edge at R = 12 cm. The peak positions at the edge and center were almost the same, but their intensities were slightly different. From the front position and intensity, it can be seen that the peak position shift of 510 cm^−1^ Si–O–Si is the result of receiving laser and environmental radiations [13]. Glass is mainly composed of SiO_4_ tetrahedrons, BO_4_ tetrahedrons, cyclic silicon metaborate groups, and a small amount of boron oxygen groups. Among them, SiO_4_ tetrahedrons are mainly connected by single non-bridged groups, and BO_4_ tetrahedrons are connected by hexaborate [14]. The connection mode of SiO_4_ tetrahedrons characterizes the degree of polymerization of the internal network of the glass. The higher the number of bridge oxygen bonds, the higher the degree of polymerization [15,16]. Figure 8 indicates more single non-oxygen bridges taking place in the center position rather than the edge, meaning that the degree of internal polymerization was not high because a large number of point defects are formed inside the glass. Many ring networks of different sizes leading to increased light absorption and decreased glass transmittance were formed.

### 3.5. EPR Analysis

**Figure 9:**
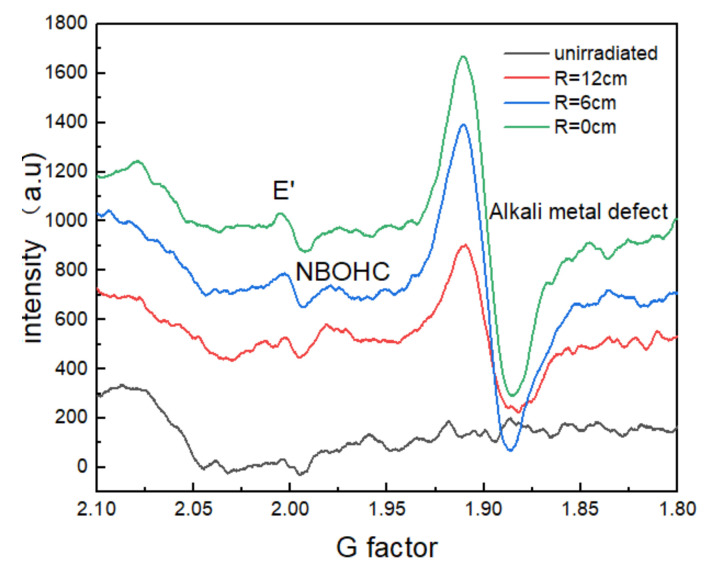
EPR spectra of nonirradiated and irradiated glass at different positions.

EPR spectroscopy qualitatively analyzes possible defects in the glass. We selected three equidistant positions from the edge to the center of the glass and compared their EPR spectra with that of the nonirradiated glass (Figure 9). According to the g-factor analysis, at three different positions, compared with the nonirradiated glass, E′ centers (≡Si, g = 2.0006) and NBOHC (g = 2.0093) appeared [17], and a non-bridge formed by the B ion group was formed in the center position. The strong signal front formed at g = 1.901 may originate from point defects formed by alkali metal impurity ions [18], such as Na^+^ and Ti^4^+ in the glass under the combined influence of laser and environmental radiation. The defect signal strength at the center position was significantly higher than the edge position, indicating that the center position had more defects than the edge position. This conclusion confirms the larger decrease in transmittance at the center position than that at the edge position.

## 4. Discussion

When the tokamak is in operation, impurities and radiation will be generated inside the device. The length of a round of experimental discharge is about 150 h. Environmental radiation has a bad influence on the glass film and glass itself, and in severe cases will directly affect the diagnostic performance. It can be seen from Figure 3 that at the edge position where the laser was not irradiated, the transmittance at 1064 nm was reduced to 94.62% due to the influence of environmental rays and impurities. After removing the film and repolishing the glass, the transmittance at 1064 nm drops to 90.23%. The transmittance difference (90.23%, compared to 91.06% of the nonirradiated glass) was very small, indicating that environmental rays and impurities affect the surface coating performance but minimally do so to the glass itself, which is consistent with the results of EPR analysis in Figure 8. The edge position was only affected by rays and impurities in the device, and the inside of the glass was less affected. If the window itself only receives radiation, after one round of experiments, the glass can be polished, coated, and reused. If the window glass receives long-term laser radiation in addition to environmental rays, and under the influence of C element impurities produced by the tokamak lower divertor, the laser will cause serious damage to the H_f_O_2_ coating, and the film performance will be greatly reduced. When high-energy photons and rays pass through the glass, a large number of point defects are induced in the glass by the laser. This leads to the increase in light absorption and the decrease in the overall transmittance curve. The long-term accumulation increased point defects inside the glass, resulting in a drop in the overall transmittance to 85.6%, which was significantly lower in the visible light band. When the film and glass were severely damaged at the same time, the transmittance of the window glass dropped to 77.61%, which already had a greater impact on the diagnostic accuracy. During the experiment, the window glass of the EAST device also assumed the role of sealing the vacuum, and the glass was inconvenient to replace so that the accuracy error of related diagnosis in the later stage of the experiment was large.

## 5. Conclusions

The optical performance of glass was studied under laser and environmental irradiations. After the single-round experiment was irradiated for about 150 h, the glass had severe transmittance due to the damage of the H_f_O_2_ coating and the defects induced by the laser inside the glass. In the position that received only environmental radiation and comprised impurities, the glass coating was more affected, while the glass itself was less affected. Summarizing the overall research results:

(1) Long-term high-frequency laser irradiation was the main cause of damage to the film and glass. Under the combined influence of the radiation and carbon impurities generated during the operation of the device, the film suffered severe ablation damage. Under the influence of device radiation and impurities, the performance of the film also decreased to a certain extent. The deposition of carbon impurities inside the EAST device strengthens the film absorption and strengthens the film damage. To reduce the damage of the film, strengthening the cleaning of carbon impurities in the divertor can be effective, extending the service life of the film.
(2) Under the combined action of radiation, impurities, and the laser, the optical performance of the glass in the middle of the experiment can no longer meet high-precision diagnostic use. It needs to be replaced when the experiment time is about 100 h to avoid influence on the later stages of the experiment.
(3) Given the electromagnetic radiation environment of the EAST device during the discharge process, choosing a film and glass with a higher laser threshold and better radiation resistance can extend the service life of the glass and restrict the damage of radiation.

## Figures and Tables

**Table 1:** Nd: YAG laser technical index

Parameter Name	Parameter Requirements
Laser wavelength	1064 nm
Repetition frequency	1–100 Hz
Output energy	3 J
RMS	<2%
Pulse width	10–12 ns
Divergence angle	<0.4 mrad
Beam pointing stability	<50 μrad
spot diameter	14 mm

**Table 2:** Tokamak lower window glass compositionol%.

Ingredient	SiO_2_	Al_2_O_3_	Na_2_O	B_2_O_3_	TiO_2_
percent	63.7	4.10	13.32	16.88	2

**Table 3:** Surface analysis results of the C element.

Number	Area (P) CPS. eV	Atomic %	Number	Area (P) CPS. eV	Atomic %
1-a	87,181.83	50.80	1-b	57,941.53	38.96
2-a	83,425.60	49.04	2-b	32,323.85	35.54
3-a	98,147.36	53.80	3-b	50,188.14	31.05
4-a	106,075.46	56.88	4-b	44,143.06	27.21
5-a	95,094.81	65.54	5-b	53,564.37	31.40

## Data Availability

The data presented in this study are available on request from the corresponding author.
